# Duration of Life

**Published:** 1874-02

**Authors:** 


### DURATION OF LIFE.

The average duration of life of man in civilized society is about thirty-three and a third years. This is called a generation, making three in a century. But there are certain localities and certain communities of people where this average is considerably extended. The mountaineer lives longer than the lowlander; the farmer than the artisan; the traveler than the sedentary; the temperate than the self-indulgent; the just than the dishonest. "The wicked shall not live out half his days," is the announcement of Divinity. The philosophy of this is found in the fact, that the moral character has a strong power over the physical; a power much more controlling than is generally imagined. The true man conducts himself in the light of Bible precepts; is "temperate in all things;" is "slow to anger;" and on his grave is written: "He went about doing good." In these three things are the great elements of human health: the restraint of the appetites; the control of the passions; and that highest type of physical exercise, "going about doing good." It is said of the eminent Quaker philanthropist, Joseph John Gurney, that the labor and pains he took to go and see personally the objects of his contemplated charities, so that none of them should be unworthily bestowed, was of itself almost the labor of one man, and he attended to his immense banking business besides; in fact, he did too much, and died at sixty. The average length of human life of all countries, at this age of the world, is about twenty-eight years. One quarter of all who die do not reach the age of seven; one half die before reaching seventeen; and yet the average of life of "Friends," in Great Britain and Ireland, in 1860, was nearly fifty-six years, just double the average life of other peoples. Surely this is a strong inducement for all to practice for themselves, and to inculcate it upon their children day by day, that simplicity of habit, that quietness of demeanor, that restraint of temper, that control of the appetites and propensities, and that orderly, systematic, and even mode of life, which "Friends'" discipline inculcates, and which are demonstrably the means of so largely increasing the average of human existence.

Reasoning from the analogy of the animal creation, mankind should live nearly an hundred years; that law seeming to be, that life should be five times the length of the period of growth; at least, the general observation is, that the longer persons are growing, the longer they live; other things being equal. Naturalists say,

| | | | | | |
|---|---|---|---|---|---|
| A dog | grows for | 2 | years, | and lives | 8 |
| An ox | " | 4 | " | " | 16 |
| A horse | " | 5 | " | " | 25 |
| A camel | " | 8 | " | " | 40 |
| Man | " | 20 | " | should live | 100 |

But the sad fact is, that only one man for every thousand reaches one hundred years. Still it is encouraging to know, that the science of life, as revealed by the investigations of the physiologist and the teachings of educated medical men, is steadily extending the period of human existence. The distinguished historian Macaulay states, that in 1685 one person in twenty died each year; in 1850, out of forty persons, only one died. Dupin says, that from 1776 to 1843 the duration of life in France increased fifty-two days annually, for in 1781 the mortality was one in twenty-nine; in 1853, one in forty. The rich men in France live forty-two years on an average; the poor, only thirty. Those who are "well to do in the world" live about eleven years longer than those who have to work from day to day for a living. Remunerative labor and the diffusion of the knowledge of the laws of life among the masses, with temperance and thrift, are the great means of adding to human health and life; but the more important ingredient, happiness, is only to be found in daily loving obeying, and serving Him "who giveth us all things richly to enjoy."

